# The Application of TD/GC/NICI–MS with an Al_2_O_3_-PLOT-S Column for the Determination of Perfluoroalkylcycloalkanes in the Atmosphere

**DOI:** 10.1007/s10337-013-2584-6

**Published:** 2013-10-15

**Authors:** Yu Ren, Hans Schlager, Damien Martin

**Affiliations:** 1 Deutsches Zentrum für Luft- und Raumfahrt (DLR), Institut für Physik der Atmosphäre, Oberpfaffenhofen, Germany; 2 Centre for Climate and Air Pollution Studies (C-CAPS), Department of Physics, National University of Ireland, Galway, Ireland

**Keywords:** TD/GC/NICI–MS, Al_2_O_3_-PLOT-S column, PFCs, Tracer

## Abstract

A modified method for the quantitative determination of atmospheric perfluoroalkylcycloalkanes (PFCs) using thermal desorption coupled with gas chromatography and detection by negative ion chemical ionization–mass spectrometry was developed. Using an optimized analytical system, a commercially available Al_2_O_3_ porous layer open tubular (PLOT) capillary column (30 m × 0.25 mm) deactivated with Na_2_SO_4_ was used for separation of PFCs. Improvements in the separation of PFCs, the corresponding identification and the limit of detection of PFCs using this method and column are presented. The method was successfully applied to determine the atmospheric background concentrations of a range of PFCs from a number of samples collected at a rural site in Germany. The results of this study suggest that the method outlined using the Al_2_O_3_-PLOT-S capillary column has good sensitivity and selectivity, and that it can be deployed in a routine laboratory process for the analysis of PFCs in the future research work. In addition, the ability of this column to separate the isomers of one of the lower boiling PFCs (perfluorodimethylcyclobutane) and its ability to resolve perfluoroethylcyclohexane offer the opportunity for single-column analysis for multiple PFCs.

## Introduction

Perfluoroalkylcycloalkanes (PFCs) are hydrocarbons normally consisting of four, five and six atom carbon rings, in which all hydrogen atoms are substituted by fluorine atoms. PFCs have been used as tracer compounds for the simulation of atmospheric transport and dispersion of pollutants for a number of decades because they are non-reactive, non-toxic, non-scavenging and non-depositing, and contribute neither to stratospheric ozone depletion nor to production of tropospheric air pollution [1–5]. They are, however, potent greenhouse gases with a high global warming potential (GWP of about 7,000) and the atmospheric lifetime of PFCs is expected to be more than 2,000 years [6]. Their background concentrations are typically a few parts per quadrillion (fL L^−1^) in the atmosphere [2–5, 7–9]. The release of minute quantities of PFCs can produce clear elevations above background and make them very suitable for model evaluation, and their global warming impact is insignificant as compared to other greenhouse gases.

To quantify these compounds, a gas chromatograph (GC) equipped with an electron capture detector (ECD) has been used in many previous studies [2–4, 7–10]. Using this method, the PFCs are detectable at the femtoliter level. However, the analytical process can suffer interferences from other compounds such as halocarbons with more abundant concentration in the atmosphere as the ECD is non-specific. To overcome this problem, a catalytic reaction was introduced to remove halocarbons from the samples prior to quantification of PFCs [2, 3, 10].

An alternative method is the use of negative ion chemical ionization (NICI)–mass spectrometry (MS) which was originally reported by Begley. et al. [11]. It was found that this technique could provide a very selective and extremely sensitive method for the determination of PFC tracers. In their research, Begley and his coworkers used a 50 m × 0.2 mm inner diameter (ID) fused silica column coated with 0.33-μm cross-linked SE-54 for the separation of PFCs. After injection of the sample, the oven temperature was held at −80 °C for 3 min by liquid nitrogen cooling to achieve minimum band broadening. However, the need for cryogenic cooling increased the complexity of the analysis. To get an ideal separation for all possible isomers of PFCs and avoid the use of liquid cryogen, different capillary columns have been tested and used in GC/NICI–MS system, such as gas–liquid wall-coated open tubular (WCOT) CP-Sil 5 CB methyl silicone column [12] and Al_2_O_3_ porous layer open tubular (PLOT) column deactivated by KCl [13]. On these columns, perfluoromethylcyclopentane (PMCP) and perfluoromethylcyclohexane (PMCH) can be resolved, but the isomers from perfluorodimethycyclohexane (PDCH) are co-eluted. In addition to this, the isomers of PDCB were not resolved well. Simmonds and the coworkers reported that all six possible isomers of PDCH can be separated from the baseline using a graphitized carbon-based PLOT capillary column [5]. However, the all isomers of PDCB co-elute on this column and this type column was no longer commercially available several years ago.

In this study, an Al_2_O_3_ porous layer open tubular (PLOT) column deactivated with Na_2_SO_4_ (Al_2_O_3_-PLOT-S) is utilized. For the analysis of perfluoroalkanes and fluorocarbons, the alumina PLOT column has been regarded as an ideal column [14] and used extensively [13, 15]. To our knowledge, no work has been reported on the use of a commercially available Al_2_O_3_-PLOT-S column for the separation of the PFCs, especially in combination with NICI–MS. This paper presents a new non-cryogenic routine analytical method for the analysis of PFCs. Using a judicious choice of temperature program, several major PFCs and their isomers can be well resolved. Compared with the results from the Carbograph PLOT, the separation of the isomers of PDCH on the column Al_2_O_3_-PLOT-S will be discussed. The analysis was carried out by an enrichment technique based on a two-step desorption procedure followed by separation and quantification by GC/MS analysis. The practical application of this method was demonstrated by quantifying the atmospheric background concentrations of PFCs from a number of samples at a rural site in Germany. These data are presented and the analytical precision is quantified.

## Experimental Section

### Instrumental Description

GC/MS analysis was performed by a TSQ GC Quantum (Thermal Fisher Scientific, USA) which can be operated in either electron ionization (EI) or chemical ionization (CI) mode. For the measurements of PFCs, mass spectrometer was operated in NICI mode.

Separations were carried out on two columns: a 30 m × 0.25 mm × 5.0 μm Al_2_O_3_-PLOT-S capillary column (HP-PLOT Al_2_O_3_ S, Agilent, USA) and a 30 m × 0.32 mm Carbograph 1 PLOT column (Lara s.r.l, Italy) provided by University of Bristol. To avoid particle shedding from the stationary phase layer of PLOT capillary column entering MS detector, a particle trap of 5 m × 0.25 mm deactivated Fused Silica column (Agilent, USA) was connected with Al_2_O_3_-PLOT-S column and a 5 m × 0.32 mm (Agilent, USA) deactivated Fused Silica column was used in tandem with the Carbograph 1 PLOT column.

The Tracer GC Ultra (Thermal Fisher Scientific, USA) temperature programs and column flow rates for both columns are shown in Table 1. High-purity helium (Alphagaz™ He, 6.0, Air Liquide) was used as carrier gas. The mass spectrometer was operated at 70 eV in negative ionization mode with methane (N55 quality, Air Liquide) as reagent gas with a flow rate of 1.5 mL min^−1^ and in either SIM (selected ion monitoring) mode: ion detection at 300, 350 and 400 amu, or SCAN mode with a range of 50–550 amu. The GC/MS interface and the source temperature were set to 190 °C.

**Table 1:** Method used in Tracer GC Ultra for two columns when TD was connected

Method description	Carbograph PLOT	Al_2_O_3_-PLOT-S
Initial temperature	35 °C	100 °C
Initial time	5 min	0.5 min
Ramp rate 1	20 °C min^−1^	30 °C min^−1^
Final temperature 1	50 °C	130 °C
Hold time 1	10 min	6.5 min
Ramp rate 2	2.5 °C min^−1^	30 °C min^−1^
Final temperature 2	65 °C	140 °C
Hold time 2	1 min	23 min
Ramp rate 3	20 °C min^−1^	20 °C min^−1^
Final temperature 3	200 °C	190 °C
Hold time 3	4 min	5 min
Carrier gas flow rate	1.5 mL min^−1^	1 mL min^−1^

### Thermal Desorption

The thermal desorption was carried out with ATD650 (PerkinElmer, USA) thermal desorber. The ATD650 is connected to the injection port of the GC through a temperature-controlled interface. Stainless steel desorption tubes used for PFC sampling (89 mm × 6.4 mm OD (outer diameter), 5 mm ID, Perkin Elmer) are filled with approximately 200 mg Carboxen 569 20–45 mesh (Supelco, USA). The focusing trap contained about 100-mg Carboxen 569 and is used for PFC enrichment. All sampling tubes and the cold trap were conditioned at 260 °C for at least 5 h under high-purity Helium flow to clean before use.

This is the two-step desorption procedure. During the primary desorption, each sample tube was heated at 250 °C for typically 20 min, and adsorbed PFCs were flushed through a 4-port valve onto the cold trap which is held at −30 °C. The flow rate for the primary desorption is at 40 mL min^−1^. After the primary desorption was finished, the 4-port valve switched simultaneously to the position of the secondary desorption. The trap is heated instantly to 260 °C and the sample is desorbed in ~3 s onto the analytical column in GC oven with a split flow of 20 mL min^−1^. The split mode injection and the rapid thermal desorption process ensure that the sample can be transferred onto the column with negligible band broadening. The temperatures of the interface and valve were both held at 200 °C.

Blank tubes are periodically checked (e.g., 6 tubes in each 20-tube batch) to ensure that they are free of PFCs before sampling. For the analysis of the ambient air samples in this work, 30-min dry purging by helium with a flow rate of 50 mL min^−1^ was applied to remove moisture before the 2-step desorption.

### Calibration

To characterize the elution of each PFC, individual and mixed PFC solutions were injected directly from the inlet of GC using similar temperature programs for the two aforementioned columns. Pure liquid PFCs were purchased from F2 Chemicals (UK). Some of the key properties of these PFCs and their purity are listed in Table 2. They were diluted to low concentration solution in isooctane (SupraSolv, Merck, Germany) before introduction to the GC.

**Table 2:** The properties of relevant PFCs

PFC names	Acronyms	Molecular formula	Molecular weight	Melting point (°C)	Boiling point (°C)	Density (g mL^−1^ at 25 °C)	Purity (%) (F_2_ Chemicals)
Perfluorodimethylcyclobutane	PDCB	C_6_F_12_	300	−32.0	45.0	1.67	
Perfluoromethylpentane	PMCP	C_6_F_12_	300	−50.0	48.0	1.707	94.7
Perfluoromethylcyclohexane	PMCH	C_7_F_14_	350	−37.0	76.0	1.788	95.3
Perfluoro-*o*-dimethylcyclohexane	*o*-PDCH^a^	C_8_F_16_	400	−22.0	102.0	1.828	88.8
Perfluoro-*m*-dimethylcyclohexane	*m*-PDCH^b^	C_8_F_16_	400	−70.0	102.0	1.828	96.6
Perfluoro-*p*-dimethylcyclohexane	*p*-PDCH^c^	C_8_F_16_	400	<−80.0	102.0	1.828	96.8
Perfluoroethylcyclohexane	PECH	C_8_F_16_	400	<−80.0	101.7	1.829	93.7

^a^The cis and trans isomers of *o*-PDCH are *oc*- and *ot*-PDCH

^b^The cis and trans isomers of *m*-PDCH are *mc*- and *mt*-PDCH

^c^The cis and trans isomers of *p*-PDCH are *pc*- and *pt*-PDCH

Gas standards are prepared at the University of Bristol and used for the measurement of the ambient samples. The detail of the preparation process can be found previously [5]. Here is the process in brief. Two gravimetrically prepared primary standard gas mixtures with a stated accuracy of ±1 % were purchased from Linde Gases Ltd., UK. Two primary standards (PFC1 and PFC2): in PFC1 20.1 ppm (μmol mol^−1^) PMCP, 19.9 ppm PDCB, 20.0 ppm PMCH and the high levels of CCl_3_F (2,296 ppm) and CCl_2_F_2_ (4,998 ppm) were contained, and in PFC2 20.3 ppm *o*-PDCH, 20.4 ppm *m*-PDCH, 20.2 ppm *p*-PDCH and the high levels of CCl_3_F (2,298 ppm) and CCl_2_F_2_ (5,001 ppm) were contained. The make-up gas was nitrogen (grade 5.0, Air Products). These standards are volumetrically diluted in a single step into a 34-L electro-polished stainless steel cylinder (Essex Industries, USA) to concentration of ~2 ppt (pmol mol^−1^). The dilution system is based on a design developed by the Scripps Institution of Oceanography (SIO).

### Sample Collection

A number of background atmospheric samples were collected at a rural site (11.264ºE, 48.074ºN), near the town of Wessling about 30 km southwest of Munich. Samples were collected onto adsorption tubes for a time interval of 40 min and a sampling flow rate of 200 mL min^−1^ using a commercially available sequential tube sampler (STS 25, PerkinElmer, USA). Before and after sampling, the flow rate through every sampling orifice in the sampler was measured with a Gillian Galibrator-2 (Scientific Instrument Services, Inc™, USA). 23 background samples were collected in March, 2011 and 19 samples from September to October, 2011. During each of sampling period, blank samples were also included in the sampling regime. After collecting, the tubes were sealed at both ends and analyzed as soon as feasible.

Previous research has concluded that the atmospheric background concentrations are independent of meteorological parameters [8]. To avoid long dry purge time for water removal, the majority of samples were collected when the weather was dry and typically the local time between 10:00 and 16:00.

## Results and Discussion

### Identification of PFCs on the Al_2_O_3_-PLOT-S Column

**Fig. 1:**
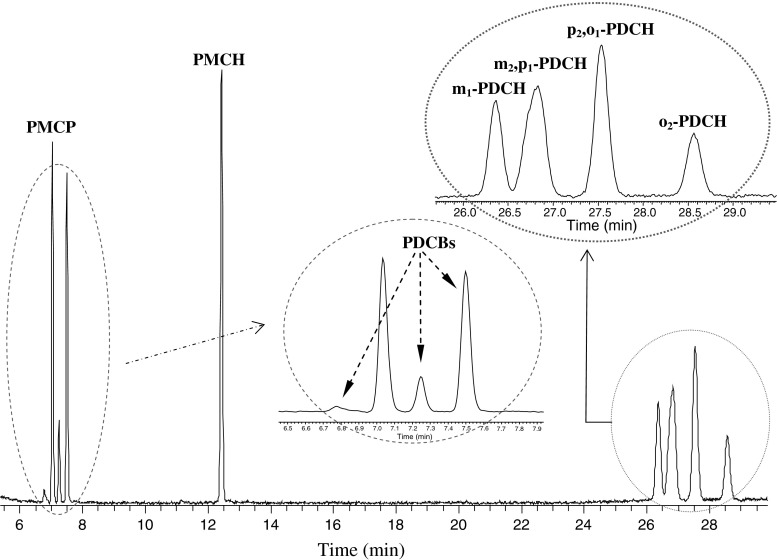
Chromatogram (in SIM mode with the selected ions of *m/z* 300, 350 and 400) of a mixed PFC gas standard diluted from PFC1 and PFC2 on the HP Al_2_O_3_-PLOT-S column

**Fig. 2:**
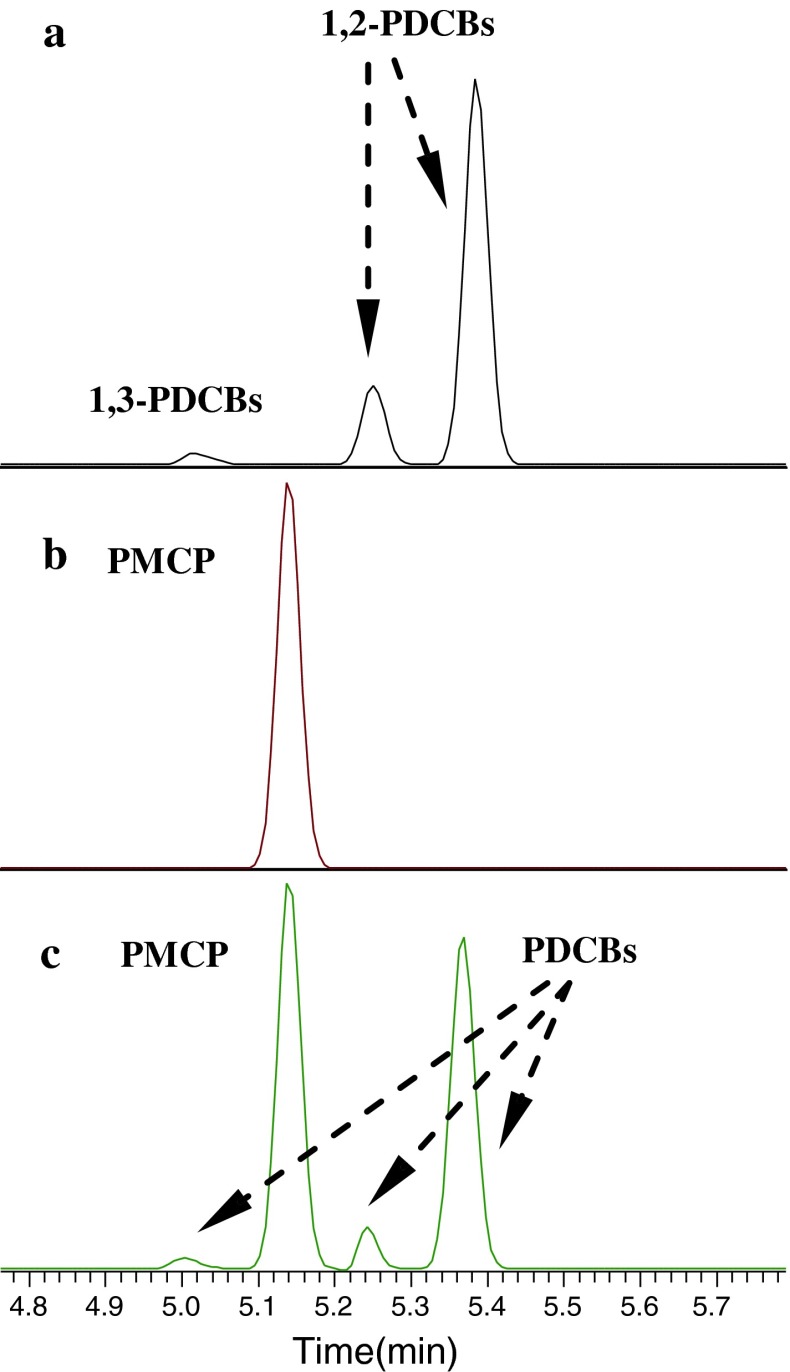
Separation of PDCB and PMCP by injecting individual PDCB (**a**), PMCP (**b**) and the mixture of PDCB and PMCP (**c**)

Due to the very similar physical properties (shown in Table 2) of some of the PFCs, such as PDCB and PMCP, and isomers of PDCH and PECH, it can be difficult to separate these PFCs effectively [5, 12, 13]. After evaluating several different capillary columns, the Al_2_O_3_-PLOT-S column was selected for these PFCs because of its good separation capability and its commercial availability. The separation of a diluted gas standard of PFCs from PFC1 and PFC2 achieved by Al_2_O_3_-PLOT-S column is shown in Fig. 1, which was detected in SIM mode with the selected ions of *m/z* 300, 350 and 400 combined for all PFCs. As can be seen, the preferred perfluorocarbon tracers PMCP and PMCH, which are normally chosen for the tracer transport and dispersion studies, show excellent resolution. The chromatogram in Fig. 1 illustrates that the isomers of PDCB have been well resolved into three peaks, which have a similar retention to PMCP (see Fig. 2). The main isomer of liquid PDCB supplied by the manufacture for the preparation of the gas standard is perfluoro-1,2-dimethylcyclobutane (1,2-PDCB). The two cis/trans isomers of 1,2-PDCB correspond to the primary and the secondary peaks in Fig. 2a. The smallest peak is perfluoro-1,3-dimethylcyclobutane (1,3-PDCB), which exists normally as an impurity in the liquid 1,2-PDCB. The separation of the cis and trans 1,2-PDCB allows the possibility of using PDCB in a tracer release experiment. However, the compositions of PDCB depend on the temperature during the production process [16, 17]. When the detailed formation conditions of PDCB are available, these cis/trans isomers of 1,2-PDCB can be further identified.

**Fig. 3:**
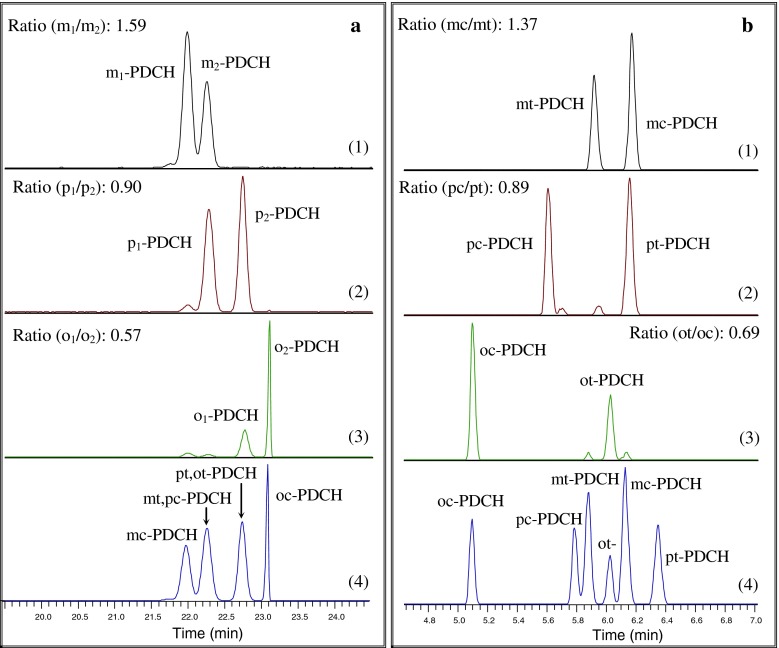
Identification of the isomers of PDCH from the individual structural isomer and mixture of PDCH liquid standards on **a** Al_2_O_3_-PLOT-S column; **b** Carbograph PLOT column. The average ratio of the integration area of *two main peaks* in every chromatogram is given in *1*–*3* with repeat injection and the *small unmarked peaks* in the chromatograms are some impurities in each individual PDCH

The six possible isomers of PDCH were resolved into four peaks as the following order *m*_1_-, (*m*_2_ and *p*_1_)-, (*p*_2_ and *o*_1_)- and *o*_2_-PDCH as shown in Fig. 1. The two co-eluted peaks come from (1) *mt* and *pc*-PDCH and (2) *pt*-PDCH and *ot*-PDCH. The average ratios of each of the three PDCH isomers integration area of peak 1/peak 2 were obtained by repeat injections (*n* = 5). These values are 1.59 (*m*_1_-/*m*_2_-PDCH), 0.90 (*p*_1_-/*p*_2_-PDCH), and 0.57 (*o*_1_-/*o*_2_-PDCH), respectively. The identification of PDCH on Al_2_O_3_-PLOT-S column was done by comparing the results from the Carbograph PLOT column through repeat injection of the same PDCHs liquid standards (*n* = 3). As expected, all these six possible isomers were separated excellently as shown in Fig. 3b. The peaks of pc- and pt-PDCHs drifted slightly as compared to their retention time in Fig. 3b (4), but they could be easily recognized from the chromatograms.

The average area ratios of *mc*-/*mt*-PDCH, *pc*-/*pt*-PDCH and *ot*-/*oc*-PDCH are 1.37, 0.89 and 0.69, respectively, which are presented in Fig. 3b (1)–(3). The ratios for isomers of PDCH displayed slightly difference from the two columns, which could be correlated with the inherent impurities and their changing elution times included in each individual PDCH and the different separation characteristics of the two columns. For example, as seen in Fig. 3a (1), there is a small peak resolved from *m*-PDCH, which should come from an impurity of PECH in the *m*-PDCH. On the column Al_2_O_3_-PLOT-S, PECH can be resolved right before the peak of *mc*-PDCH, which will be shown in the chromatogram when an atmospheric background sample was analyzed. However, PECH co-eluted with *mt*-PDCH on Carbograph PLOT column. This is likely the reason that the ratio of *mc*/*mt*-PDCH on Al_2_O_3_-PLOT-S is a little larger than that on Carbograph. As a result, the Al_2_O_3_-PLOT-S column shows its potential advantage for the analysis of PECH and its use in potential tracer experiments and represents a significant enhancement in the range of PFCs that could be used in tracer experiments.

### Measurement of Atmospheric Background Concentrations of PFCs

To demonstrate the feasibility of the analytical method, 42 air samples were collected at a rural site (Oberpfaffenhofen) in Germany and quantified with either of the two columns investigated. 23 samples were analyzed on the Carbograph PLOT column, while the remaining 19 were analyzed on Al_2_O_3_-PLOT-S column. Precision, linearity and response curves for the two columns are summarized in Table 3. For the measurement of the PFCs, all the analytes were detected in SIM mode with the selected ions of *m/z* 300, 350 and 400. The calibration curves of all PFCs are linear at least two orders of magnitude for external standard calibration. The sensitivity of the GC/MS in NICI-SIM mode was quantified on the two columns by introducing predefined quantities of the PFC standards. The limit of detection (LOD) is defined as the PFC volume (fL) having a three times signal-to-noise ratio and shown in Table 3. During the analytical cycle, PFC standard analysis was carried out every two or three air samples analyzed. The precision of these analyses with the relative standard deviation (%) is also shown in Table 3.

**Table 3:** The validation data of the analytical method

PFCs	Carbograph PLOT					Al_2_O_3_-PLOT-S				
	Calibration curve	*r* ^2^	Linearity range (fL L^−1^)	LOD (fL)	Precision (*n* = 8)	Calibration curve	*r* ^2^	Linearity range (fL L^−1^)	LOD (fL)	Precision (*n* = 10)
PDCB	*y* = 5,112.9*x* + 17,407	0.9990	4–4,800	0.9	8.4	*y* = 2,307.5*x* + 10,771	0.9958	4–3,700	1.2^a^	2.8^a^
PMCP	*y* = 4,234.8*x* + 11,236	0.9994	4–4,800	0.6	6.6	*y* = 3002*x* + 14,243	0.9985	4–4,800	1.2	1.7
PMCH	*y* = 3,564.2*x* + 10,509	0.9993	9–9,600	1.2	8.4	*y* = 1,844.1*x* + 7,941.9	0.9987	9–9,600	2.5	1.8
*oc*-PDCH	*y* = 3,753.8*x* + 7,607.9	0.9990	6–2,900	1.7	7.0	*y* = 2,251.6*x* + 6,589.7	0.9979	12–2,900	3.2	3.3
*pc*-PDCH	*y* = 4,964.6*x* + 11,165	0.9992	5–3,500	1.5	8.4	*y* = 2,942.8*x* + 15,690 (*mt*,*pc*-PDCH)	0.9983	10–5,100	3.0	4.4
*mt*-PDCH	*y* = 7,050.9*x* + 9,241.3	0.9986	3–1,600	0.9	7.4					
*ot*-PDCH	*y* = 6,251.1*x* + 4,769.7	0.9989	6–1,100	1.2	8.8	*y* = 4,200.9*x* + 15,528 (*pt*,*ot*-PDCH)	0.9984	10–3,800	1.8	2.2
*pt*-PDCH	*y* = 5,853.4*x* + 13,433	0.9984	4–2,600	1.1	7.8					
*mc*-PDCH	*y* = 6,347.1*x* + 10,831	0.9989	12–2,600	1.2	6.7	*y* = 3,728.8*x* + 5,618.9	0.9990	12–2,600	3.2	4.9

The precision analysis is expressed as percentage relative standard deviation

^a^The third peak of PDCB in Fig. 1

**Fig. 4:**
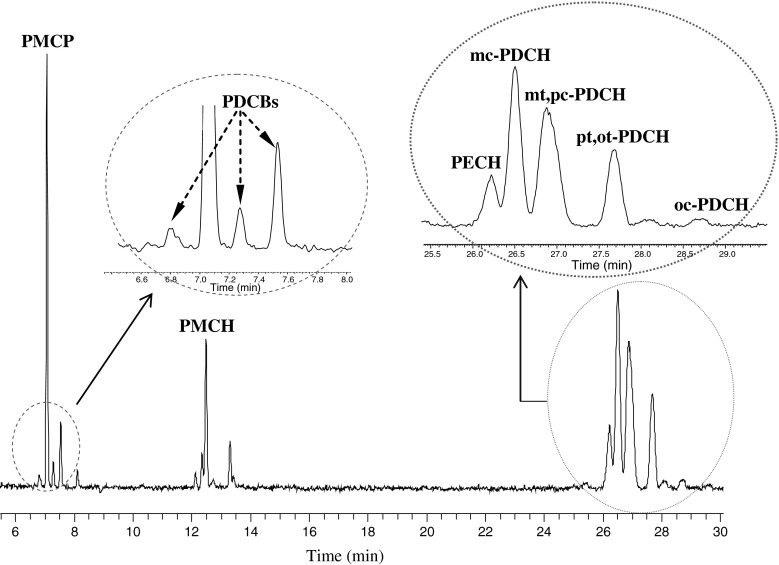
Chromatogram (in SIM mode with the selected ions of *m/z* 300, 350 and 400) of an ambient sample analyzed on Al_2_O_3_-PLOT-S capillary column

Figure 4 shows a typical chromatogram of an air sample separated on Al_2_O_3_-PLOT-S column. As shown, a number of the atmospheric PFCs are well resolved. In addition, several other PFC peaks were also obtained. The peak eluting before the peaks of PDCH is PECH, which was determined by injecting the individual standard solution. The ability of this column to separate PECH represents a significant improvement of the Al_2_O_3_-PLOT-S over the Carbograph PLOT column. Identifying unknown compounds through the use of individual standards will be the subject of the future studies.

Table 4 lists the background concentrations of PFCs as determined by the two columns, as well as those from previous background studies. As shown in Table 4, the PFC concentrations analyzed from two analytical columns are very similar and they are consistent with previous measurements. This result demonstrates the feasibility of the proposed method for the analysis of PFCs. Levels of some PFCs are slightly lower than those reported from previous measurements, which may be caused by different sampling times and locations. As well, different analytical system with different columns, elution with other perfluorocarbon tracer species may result in some discrepancies among these studies. In addition, different calibration scales may also contribute to these differences.

**Table 4:** Background concentrations of PFCs in the atmosphere in mean values (fL L^−1^) ± standard deviation

	PDCB	PMCP	PMCH	*oc*-PDCH	*pc*-PDCH	*mt*-PDCH	*ot*-PDCH	*pt*-PDCH	*mc*-PDCH
OP-Carbo^a^	0.85 ± 0.11	6.84 ± 1.43	4.56 ± 0.40		3.36 ± 0.40	6.06 ± 0.50		2.82 ± 0.28	7.51 ± 0.55
OP-Al_2_O_3_^b^	0.57 ± 0.05^c^	6.41 ± 0.79	4.10 ± 0.35		9.25 ± 0.99^d^		3.03 ± 0.46^e^		7.06 ± 0.43
ANATEX 1987 [7]	2.09 ± 0.43	2.09 ± 0.43	3.60 ± 0.05	0.40 ± 0.03		11.70 ± 2.57		4.34 ± 0.32	
ETEX, Austria, 1994 [8]	0.63 ± 0.18	5.24 ± 1.03	5.90 ± 1.64	0.98 ± 0.48				5.37 ± 1.85	
New Jersey, Rural, 1996 [3]		4.15 ± 0.25	3.84 ± 0.17	0.34 ± 0.02		12.80 ± 0.41^f^			8.41 ± 0.26
Mace Head 2001 [5]	2.81 ± 0.22	6.43 ± 0.14	5.46 ± 0.31	0.92 ± 0.11	6.13 ± 0.48	8.52 ± 0.18	0.77 ± 0.10	4.78 ± 0.21	10.73 ± 0.33
Florida, Remote 2006 [4]	3 ± 0.4	8 ± 0.6	7 ± 0.9	1 ± 0.4				8 ± 0.8	

^a^23 samples were analyzed on the Carbograph PLOT column in this study

^b^19 samples were analyzed on the Al_2_O_3_-PLOT-S column in this study

^c^The value represents the concentration of the third peak of PDCB in Fig. 4

^d^The value represents the total concentration of *pc*- and *mt*-PDCH

^e^The value represents the total concentration of *ot*- and *pt*-PDCH

^f^The value represents the total concentration of *mt*- and *pt*-PDCH

In the long-term perspective, the usage of PFCs should be controlled carefully due to their high global warming potentials. However, the measurement results presented here illustrate clearly that PFC concentrations in the atmosphere are still extremely low. Watson et al. [4] estimated that the present total PFC radiative forcing is approximately 2 × 10^−5^ Wm^−2^. Accordingly, a PFC release into the atmosphere of 30 kg during a tracer experiment represents a negligible greenhouse contribution (~1.5 × 10^−10^ Wm^−2^).

## Conclusions

A feasible analytical method (TD/GC/NICI–MS) in conjunction with a commercial HP Al_2_O_3_-PLOT-S column for the measurement of atmospheric PFCs is described. The resolution of the major PFCs has been improved under the present analytical conditions with especially good separation of the three major isomers of PDCB. Four peaks of the isomers of PDCH have been separated well and identified as compared to the results from the Carbograph PLOT column. The excellent peak separation suggests that isomers of PDCB and PECH could be identified and measured on Al_2_O_3_-PLOT-S column when suitable standards are available. This separation capability would facilitate a choice of tracer for multiple PFC release which could be quantified with single-column analysis. On the basis of the data presented here, the background concentrations of PFCs are still extremely low and suitable for the use in the tracer dispersion studies. The method will be used for the measurement of perfluorocarbon tracer in the future.
